# Evaluation of the Dislodgement Resistance of a New Pozzolan-Based Cement (EndoSeal MTA) Compared to ProRoot MTA and Biodentine in the Presence and Absence of Blood

**DOI:** 10.1155/2019/3863069

**Published:** 2019-05-09

**Authors:** Alireza Adl, Nooshin Sadat Shojaee, Negar Pourhatami

**Affiliations:** ^1^Department of Endodontics, Biomaterials Research Center, School of Dentistry, Shiraz University of Medical Sciences, Shiraz, Iran; ^2^Department of Endodontics, School of Dentistry, Shiraz University of Medical Sciences, Shiraz, Iran; ^3^Postgraduate Student of Endodontics, Department of Endodontics, School of Dentistry, Shiraz University of Medical Sciences, Shiraz, Iran

## Abstract

**Introduction:**

This *in vitro* study investigated the dislodgement resistance of EndoSeal MTA, a new pozzolan-containing calcium silicate-based material, in comparison with ProRoot MTA and Biodentine in the presence and absence of contamination with blood.

**Methods:**

Standard furcal perforations were created in 180 human mandibular first molars. The teeth were randomly allocated to 12 groups of 15 each. ProRoot MTA, Biodentine, and EndoSeal MTA were used to repair the perforations. In half of the samples, the walls of the perforated areas were contaminated with blood, whereas saline was injected into the other half. A push-out test was performed using a universal testing machine after 24 hours or 7 days. To evaluate failure patterns, the samples were split into half and were examined under a stereomicroscope at a 20x magnification. Data were analyzed using three-way analysis of variance, Tukey test, and Student's *t*-test.

**Results:**

At both time intervals and in the presence and absence of contamination with blood, ProRoot MTA and Biodentine had significantly higher retention values than EndoSeal MTA (*p* < 0.001). Contamination with blood had no effect on EndoSeal MTA; however, it negatively affected the dislodgement resistance of Biodentine at 24 hours and ProRoot MTA at both time intervals (*p* < 0.05). Time significantly affected only the bond strength of the uncontaminated groups (*p* > 0.001). The most common type of failure was mixed for ProRoot MTA and Biodentine, whereas it was cohesive for EndoSeal MTA.

**Conclusions:**

ProRoot MTA and Biodentine showed higher values of bond strength than EndoSeal MTA and may thus be better options for the repair of root perforations.

## 1. Introduction

Root perforations are communications between root canal systems and external root surfaces [1]. Although perforations can be pathological, resulting from caries or resorptive defects, most of them occur iatrogenically during and after root canal treatments [2]. Root perforations are among the major causes of endodontic failures, and if not diagnosed quickly and repaired properly, they may ultimately lead to loss of the involved teeth [3–5].

Perforations of the cervical and furcal areas of the teeth have a poor prognosis, primarily because of their close proximity to supracrestal attachments that can lead to periradicular breakdown and bone loss [6, 7]. Therefore, immediate sealing of root perforations is essential for improving the prognosis of the affected teeth and for preventing the progression of the inflammatory process in periodontal tissues over time [8].

Mineral trioxide aggregate (MTA) has been suggested as the material of choice for root perforation repair because it meets many of the ideal properties of an endodontic repair material, such as good sealing ability, biocompatibility, antimicrobial activity, and hydrophilic behavior [9–11]. Nevertheless, long setting time, difficult handling, and the risk of tooth discoloration are some disadvantages of this material [12–14]. To overcome these imperfections, Biodentine (Septodont, Saint Maur des Fosses, Cedex, France) has been recently introduced as an alternative to MTA. It has superior properties, such as higher microhardness, shorter setting time, better sealing ability, and higher compressive strength than MTA [15, 16]. Some recently published case reports have demonstrated the ability of Biodentine to successfully repair root perforations [17, 18].

Lately, EndoSeal MTA (Maruchi, Wonju, South Korea), a material based on pozzolan cement with excellent physical and biological properties of MTA, has also been presented to the market. EndoSeal MTA is available in the form of a premixed, preloaded paste in an air-tight syringe, and according to the manufacturer, it has various clinical applications, including obturation of root canals and repair of root perforations. It is composed of calcium silicate, calcium aluminate, calcium aluminoferrite, and calcium sulfate [19]. During the setting process of EndoSeal MTA, pozzolan cement, which is a siliceous or siliceous-aluminous material, chemically reacts with calcium hydroxide in the presence of moisture to form compounds with adequate cementitious properties that may contribute to the effective flow of the substance, sufficient working consistency, and a reduced setting time up to approximately 4 minutes [20].

Previous studies have demonstrated several characteristics of EndoSeal MTA, including its favorable physical properties [21], minimal discoloration [22], satisfying biocompatibility [23], adequate resistance to bacterial microleakage [24], and the ability to promote dentinal tubule biomineralization [20]. However, to date, no study has compared its resistance to dislodgement with that of other biomaterials conventionally used to repair root perforations.

Moreover, materials used for the repair of perforations may get contaminated with blood during placement or setting. Several studies have confirmed that contamination with blood can adversely affect the retention characteristics of calcium silicate-based cements (CSCs) [25, 26]. Therefore, the present study is aimed at investigating the dislodgement resistance of this new pozzolan-containing calcium silicate-based material in comparison with ProRoot MTA and Biodentine in the presence and absence of contamination with blood.

## 2. Materials and Methods

Totally, 180 human extracted mandibular first molars were used in the present study. All selected teeth had mature apices with no signs of cracks, carious lesions, fused roots, size or shape anomalies, or previous root canal treatments. All procedural steps were performed by a single operator. After soft tissue remnants were removed from the external teeth surfaces, they were kept in 0.5% chloramine-T solution until use. The teeth were decoronated at the cementoenamel junction using a water-cooled diamond disc and were then mounted in acrylic molds, leaving a 3 mm space under the furcal area for subsequent placement of Gelatamp (Roeko/Coltène/Whaledent, Langenau, Germany), which would act as a matrix for packing materials during perforation repair. Simulated furcation perforations were created using a #1.2 cylindrical bur (diameter: 1.2 mm), perpendicular to the furcal floor and parallel to the long axes of the teeth. The perforations were then enlarged up to a diameter of 1.3 mm with two complete passes of a #5 Gates Glidden drill (Mani, Tochigi, Japan). A periodontal probe was used to measure the depth of the perforated area to reach a height of 2 mm in all the samples. Samples with a depth of less than 2 mm were excluded, whereas those with a greater dentin thickness were ground down using a diamond disc. All samples were then rinsed with saline to remove any debris produced during the procedure.

The samples were randomly divided into 12 experimental groups of 15 each based on the perforation repair material (Table 1), the timing of the push-out test, and the presence or absence of blood contamination. Before the placement of the reparative materials, using a condenser, pieces of Gelatamp were packed under the furcation area of all samples.

In half of the samples, the perforation sites were contaminated with blood. For this purpose, a 27-gauge syringe was used to inject blood into the perforated furcal area. The blood was provided by one of the researchers, in accordance with the ethical principles of Declaration of Helsinki for medical research involving human subjects [27]. Excess blood was removed using paper points (size: 45; taper: 0.02) without any contact with the perforation walls. In the remaining samples, instead of blood, normal saline was injected into the perforations before placing the repair materials.

In groups 1–4 and 5–8, ProRoot MTA (Dentsply Tulsa Dental, Johnson City, TN, USA) and Biodentine, respectively, were used to repair the perforations. Both materials were mixed following the manufacturer's instructions and were packed into the perforation sites using a plastic instrument.

Groups 9–12 were repaired using EndoSeal MTA. This material is provided as a premixed paste preloaded into a syringe. EndoSeal MTA was therefore injected into the perforation area using disposable needle tips. All samples were subsequently wrapped in pieces of gauze soaked in phosphate-buffered saline and were placed in closed containers in an incubator with 100% relative humidity at 37°C for 1 or 7 days.

### 2.1. Push-Out Test

The push-out test was conducted using a universal testing machine (Z050, Zwick/Roell, Ulm, Germany). The reparative materials in the perforated areas were subjected to a vertical load at a crosshead speed of 1 mm/min with a 1 mm diameter cylindrical stainless steel plunger until debonding occurred. The maximum force applied to the materials before dislodgement was recorded in newtons (N). The push-out bond strength was calculated in megapascals (MPa) using the following formula:

(1) $$\begin{array}{c} \text{Bond strength }\left(\text{MPa}\right)=\frac{\text{Force needed to dislodge the material}}{3.14\times \text{diameter of the perforation site}\times \text{height of perforation}}. \end{array}$$

### 2.2. Evaluation of Failure Patterns

**Figure 1:**
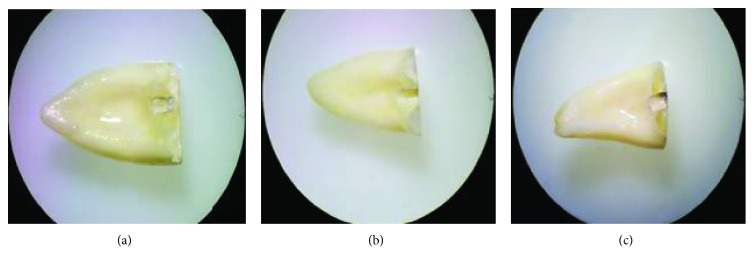
Patterns of bond failure: (a) mixed, (b) adhesive, and (c) cohesive.

After the push-out test, the samples were perpendicularly split into half and were examined under a stereomicroscope (BS-3060C, BestScope, Beijing, China) at a 20x magnification to determine the mode of failure. The mode of bond failure was categorized as follows [28]: adhesive, at the dentin-material interface; cohesive, within the material; and mixed failure, a combination of adhesive and cohesive failures (Figure 1).

### 2.3. Statistical Analysis

Three-way analysis of variance (ANOVA) was used to evaluate the significance of the effect of the restorative material type, contamination with blood, and the timing of the push-out test. One-way ANOVA, Tukey test, and Student's *t*-test were used for pairwise comparisons. Statistical analyses were performed using the SPSS 22.0 (SPSS for Windows; SPSS Inc., IBM, Chicago, IL, USA) at a significance level of 0.05.

## 3. Results

The mean and standard deviations of the push-out bond strength values in each group are listed in Table 2. Interaction effects were observed between the repair materials with respect to time (*p* = 0.001) and contamination with blood (*p* = 0.001).

At both time intervals and in the presence and absence of blood, Biodentine and ProRoot MTA had significantly higher retention values than EndoSeal MTA (*p* < 0.05). Moreover, Biodentine had higher bond strength values than ProRoot MTA at the 24-hour interval (*p* < 0.05).

Contamination with blood did not affect the EndoSeal MTA samples (*p* > 0.05); however, it negatively affected the dislodgement resistance of Biodentine samples after 24 hours (*p* = 0.009) and ProRoot MTA samples at both 24 hours (*p* = 0.02) and 7 days (*p* = 0.002).

Time exerted significant effects only on the bond strength of the uncontaminated tested materials (*p* < 0.05). When the time was increased from 24 hours to 7 days, the bond strength values increased in the ProRoot MTA (*p* = 0.005) but decreased in Biodentine (*p* = 0.008) and EndoSeal MTA (*p* = 0.021) samples.

The most common failure mode at both debonding times was mixed for Biodentine and ProRoot MTA and cohesive for EndoSeal MTA.

## 4. Discussion

The bond strength of materials used for the repair of root perforations is representative of their resistance to displacement by the mechanical forces of condensation of restorative materials and functional pressures [25, 29]. Remaining in place under these disturbing forces, particularly in the presence of body fluids or contamination with blood, has a significant effect on the prognosis of the affected teeth [25]. To the best of our knowledge, this study was the first to evaluate the push-out bond strength of EndoSeal MTA compared with that of conventional reparative materials. Effects of time and contamination with blood were also assessed in this study.

The results of the present study indicated that Biodentine and ProRoot MTA had significantly higher bond strength than EndoSeal MTA. This finding might be explained by the fact that in comparison with other CSCs, pozzolan-based materials are associated with a significantly lower Ca-releasing ability and Ca/P ratio of the apatite-like crystalline precipitates [30]. This explanation is supported by the fact that the concentration of available ions affects the nucleation and growth of the apatite layer [31]. On the other hand, a recent study has reported that the push-out bond strength of the original pozzolan-based cement (EndoCem MTA) is comparable to that of ProRoot MTA [32]. This might suggest that the pozzolanic reaction which causes lower Ca release is not a primary reason for the low dislodgement resistance of EndoSeal MTA found in the current study. However, it should be kept in mind that the particle size distribution in EndoSeal MTA is not the same as EndoCem MTA [33]. Moreover, EndoSeal MTA is premixed with nonaqueous but water-miscible carriers and, contrary to powder forms of MTA, uses only the environmental moisture to initiate and complete the setting reaction [20]. These differences might predispose EndoSeal MTA to lower dislodgement resistance.

Based on these results, Biodentine exhibited significantly higher bond strength at the 24-hour interval than ProRoot MTA. However, after 7 days, no significant differences were observed between the dislodgement resistances of Biodentine and ProRoot MTA. This outcome corroborates with the study conducted by Aggarwal et al. who compared the bond strength of Biodentine and ProRoot MTA at the same time intervals [34]. This finding can be explained by the known prolonged setting time and maturation process of MTA. The prolonged maturation process of ProRoot MTA causes the material to attain its maximum strength after 24 hours [10].

Contamination with blood had a detrimental effect on the bond strength of Biodentine after 24 hours, and for ProRoot MTA, it had an adverse effect at both time points. These findings corroborate with the results of several previous studies [25, 26, 34–36] that confirmed that contamination with blood caused a reduction in the bond strength of CSCs to dentin. The negative effect of blood has been attributed to the presence of the different cells and proteins it contains, which can simply clog the dentinal tubules and create gaps between the reparative materials and the dentinal walls [37, 38]. The clinical implication of this finding is that bleeding should be controlled before repair of perforations with these materials. However, in the present study, EndoSeal MTA dislodgement resistance was not affected by the presence of blood, probably because of its short setting time (4 minutes), which causes the material to reach its maximum strength before any environmental conditions, such as blood exposure, can negatively influence it. Nevertheless, it should not be assumed that EndoSeal MTA is the material of choice for repair of root perforations in clinical situations where it is difficult to control bleeding, because our results showed that ProRoot MTA and Biodentine, although negatively affected by contamination with blood, had higher bond strength than EndoSeal MTA.

The present study showed that time did not affect the bond strength of the blood-contaminated samples of all tested materials. A previous study evaluating the effects of blood exposure on the push-out bond strength of four CSCs at different time intervals reported similar findings for Biodentine but not for ProRoot MTA [35]. Contrary to the blood-contaminated groups in this study, the retention values of the uncontaminated samples of the three tested materials were significantly affected by time. According to these results, significantly greater force was required to displace uncontaminated ProRoot MTA samples after 7 days than that required after 24 hours. This finding was consistent with the results of the former studies [25, 26, 35]. MTA has an extended setting time [10], and at the 24-hour interval, it may have had not reached its maximum strength because of the incomplete setting reaction. By contrast, Biodentine and EndoSeal MTA, which have shorter setting times, revealed lower retention values when the time was increased from 24 hours to 7 days. Regarding Biodentine, this finding does not agree with those of the previous studies that reported no change [35] or an increase [34, 39] in the dislodgement resistance over time. Different methodologies, including different debonding times and surface treatments of the samples (keeping or removing the smear layer), may explain the inconsistencies of these results. Regarding EndoSeal MTA, it was not possible to compare our findings with those of the previous studies, because the current study is the first to evaluate this subject.

The dominant bond failure for ProRoot MTA and Biodentine in this study was a mixed failure. This finding is consistent with the results of some previous studies [26, 40] but in contrast with some others who reported that the bond failures were mainly adhesive [35, 41]. These discrepancies might be attributed to the different experimental setups of different studies. Contrary to ProRoot MTA and Biodentine, the most frequent pattern of failure for EndoSeal MTA was cohesive. This finding is supported by a previous study that reported direct tubular penetration of EndoSeal MTA and also further formation of apatite crystals in the dentinal tubules [20]. In other words, the cohesive strength within this material is weaker than its bond to dentin.

## 5. Conclusion

Within the limitations of this *in vitro* study, ProRoot MTA and Biodentine showed higher values of bond strength to root dentin than EndoSeal MTA and may serve as better options for the repair of furcal perforations. When Biodentine is used for the repair of perforations, the final restoration should be placed 24 hours later; however, in the case of ProRoot MTA, the final restoration should be postponed for 7 days. It is also recommended that bleeding at the perforation site should be controlled before placement of these reparative materials.

## Figures and Tables

**Table 1:** Materials used in this study.

Materials	Compositions
ProRoot MTA	Calcium silicate, calcium sulfate, tricalcium aluminate, calcium oxide, iron oxide, and bismuth oxide

Biodentine	Calcium silicate, calcium carbonate, calcium oxide, zirconium oxide, iron oxide, hydrosoluble polymer, and calcium chloride

EndoSeal MTA	Calcium silicate, calcium aluminate, calcium aluminoferrite, calcium sulfate, radiopacifier, and thickening agent

**Table 2:** Study groups, the mean ± standard deviation of push-out bond strength, and percentages of each failure mode in experimental groups.

Group	Material	Blood contamination	Debonding interval	Bond strength (MPa)	Failure mode (%) (*A*, *C*, *M*)
1	ProRoot MTA	+	24 hours	2.34 ± 1.28	22, 14, 64
2	ProRoot MTA	−	24 hours	3.92 ± 1.61	38, 8, 54
3	ProRoot MTA	+	7 days	3.37 ± 1.19	36, 7, 57
4	ProRoot MTA	−	7 days	8.02 ± 3.91	34, 13, 53
5	Biodentine	+	24 hours	6.02 ± 2.33	41, 6, 53
6	Biodentine	−	24 hours	9.46 ± 3.40	31, 19, 50
7	Biodentine	+	7 days	4.40 ± 2.23	27, 7, 66
8	Biodentine	−	7 days	6.10 ± 2.01	27, 13, 60
9	EndoSeal MTA	+	24 hours	1.14 ± 0.38	12, 48, 40
10	EndoSeal MTA	−	24 hours	1.37 ± 0.67	7, 53, 40
11	EndoSeal MTA	+	7 days	0.92 ± 0.34	0, 53, 47
12	EndoSeal MTA	−	7 days	0.84 ± 0.39	0, 66, 34

A: adhesive; C: cohesive; M: mixed; MTA: mineral trioxide aggregate.

## Data Availability

The data used to support the findings of this study are available from the corresponding author upon request.
